# Effectiveness and safety analysis of titanium mesh grafting versus bone grafting in the treatment of spinal Tuberculosis: a systematic review and meta-analysis

**DOI:** 10.1186/s12893-023-02283-1

**Published:** 2023-12-12

**Authors:** Fangfang Deng, Bo Chen, Huali Guo, Qingqing Chen, Feifan Wang

**Affiliations:** The First College of Clinical Medical Science, China Three Gorges University, Yichang Central People’s Hospital, Yichang Hubei, 443000 China

**Keywords:** Spinal Tuberculosis, Titanium Mesh Grafting, Bone grafting, Surgical Treatment, Meta-analysis

## Abstract

**Background:**

To systematically assess the safety and effectiveness of titanium mesh grafting compared with bone grafting in the treatment of spinal tuberculosis.

**Methods:**

Electronic databases, including PubMed, Embase, Web of Science, and Cochrane Library, were searched from their inception until April 2023. The outcome indicators for patients treated with titanium mesh grafting or bone grafting for spinal tuberculosis include surgical duration, intraoperative blood loss, graft fusion time, American Spinal Injury Association (ASIA) Spinal Cord Injury Grade E assessment, VAS score, lumbar pain score, post-graft kyphotic angle, and postoperative complications. The Newcastle-Ottawa Scale (NOS) and the Grading of Recommendations Assessment, Development, and Evaluation (GRADE) approach were used for quality assessment and evidence grading of clinical studies. Funnel plots and Begg’s test were employed for bias assessment.

**Results:**

A total of 8 studies were finally included, comprising 523 patients, with 267 cases of titanium mesh fixation and 256 cases of bone grafting. The meta-analysis showed no significant statistical differences in surgical duration (Weighted Mean Difference (WMD) = -7.20, 95% Confidence Interval (CI): -28.06 to 13.67, P = 0.499), intraoperative blood loss (WMD = 16.22, 95% CI: -40.62 to 73.06, P = 0.576), graft fusion time (WMD = 0.97, 95% CI: -0.88 to 2.81, P = 0.304), ASIA Spinal Cord Injury Grade E assessment (Relative Risk (RR) = 1.03, 95% CI: 0.97 to 1.09, P = 0.346), and overall complications (RR = 0.87, 95% CI: 0.49 to 1.55, P = 0.643). Differences in VAS score, ODI lumbar pain score, and post-graft kyphotic angle between the titanium mesh grafting group and the bone grafting group were not significant within the 95% CI range. The rate of postoperative implant subsidence was slightly lower in bone grafting than in titanium mesh grafting (RR = 9.30, 95% CI: 1.05 to 82.22, P = 0.045).

**Conclusions:**

Both bone grafting and titanium mesh grafting are effective and safe for the surgery, with no significant statistical differences in the results. Considering the limitations of the present study, large-scale randomized controlled trials are warranted to further verify the reliability of this finding.

**Supplementary Information:**

The online version contains supplementary material available at 10.1186/s12893-023-02283-1.

## Background

Osteoarticular tuberculosis is one of the most common forms of extrapulmonary tuberculosis, notorious for its alarmingly high rates of deformity, disability, and recurrence [1]. Spinal tuberculosis is the most prevalent type of osteoarticular tuberculosis, with a paraplegia rate reaching 10%. Though anti-tuberculosis remedies are traditionally prioritized for spinal tuberculosis management, relying solely on this treatment may lead to complications such as spinal cord injury and kyphotic deformities [2]. In case of persistent localized pain unresponsive to anti-tuberculous therapy, spinal cord compression and sensorimotor deficits induced by expansive paravertebral abscesses, and delayed paralysis due to severe spinal angular loss, spinal instability, and hyperkyphotic deformities, surgical intervention becomes imperative. Surgical procedures facilitate the eradication of tuberculous lesions, alleviate neural compression, rectify deformities, and restore spinal stability, thus reducing the risk of complications. Therefore, surgical treatment remains an indispensable strategy for managing spinal tuberculosis [3–5]. However, postoperative non-union and/or high recurrence rates, ranging from 13 to 26%, remain intractable challenges. These are closely associated with bone defects resulting from spinal tuberculosis mycobacterial infections [6]. Due to the intricate anatomy of the spine, its unique biomechanics, and its location at stress-concentration zones, the choice of grafting material between vertebrae demands a high degree of stability restoration. The challenge lies in effectively reconstructing the anterior defect following lesion removal. XU et al. [7] posited that both iliac bone graft and sternum handle graft can effectively repair anterior column defects caused by thorough tuberculosis lesion removal, with autografts possibly resulting in fewer donor-site complications. According to a multicenter retrospective study by YANG et al. [8], allograft bone grafting is both safe and effective in spinal tuberculosis surgery. While the fusion time of allograft bone might be slightly longer than autografts, solid fusion can be achieved within 9–18 months. In spinal tuberculosis surgery, allografts can potentially replace autografts. Previous studies have indicated that titanium mesh offers superior anterior column support and has a lower rate of kyphotic angle loss, especially beneficial for patients with osteoporosis and inferior iliac bone quality [9–16]. With the advancement of biomaterials and bone tissue engineering, a variety of grafting materials are now employed in the surgical treatment of spinal tuberculosis. However, the choice between titanium mesh and bone grafting remains controversial [17–20]. The aim of this study is to utilize systematic review methodologies to compare the effectiveness and safety of titanium mesh grafting versus bone grafting in treating spinal tuberculosis.

## Methods

This study has been registered with the international registration platform PROSPERO (CRD 42,023,415,716).

### Data sources and search strategy

Searches were conducted in PubMed, Embase, Web of Science, and Cochrane Library using the search terms “Tuberculosis, Spinal”, “Spinal Tuberculosis”, “Spinal Tuberculosis”, “Tuberculosis, Spinal”, “Pott’s Disease”, “Disease, Pott’s”, “Potts Disease”, “Pott Disease”, “Disease, Pott”, “Pott’s Paraplegia”, “tuberculosis of spine”, “thoracolumbar tuberculosis”, “lumbar tuberculosis”, “thoracic tuberculosis”, “thoracic and lumbar tuberculosis”, “titanium mesh”, “Titanium mesh cage”, “titanium mesh cage reconstruction”, “titanium mesh cages”, “Titanium mesh cages bone fusion”, “bone transplantation”, and “bone graft”. The search covered the period from the inception of each database until April 2023.

Two researchers (G.H.L. and C.Q.Q.) screened the studies for eligibility after searching articles. For any discrepancies, a third researcher (W.F.F.) was consulted for the final decision.

### Inclusion criteria and exclusion criteria

#### Inclusion criteria

The inclusion criteria were designed based on the PICO standards:

P (Patients): Patients, both male and female, with spinal tuberculosis undergoing titanium mesh cage bone grafting or bone grafting surgery.

I (Intervention): Spinal tuberculosis surgery utilizing titanium mesh grafting.

C (Control): Spinal tuberculosis surgery utilizing bone grafting.

O (Outcome): Comparing the effectiveness and safety of titanium mesh cage bone grafting and bone grafting.

#### Exclusion criteria

Reviews, animal experiments, cross-sectional studies; cases of secondary surgery; studies with metastatic or intraspinal tumors, metabolic bone disease, other bacterial infections, etc.; missing data; non-English literature; duplicate publications.

#### Outcome indicators

outcome indicators include surgical duration, intraoperative blood loss, graft fusion time, American Spinal Injury Association (ASIA) Spinal Cord Injury Grade E assessment, VAS score, lumbar pain score, post-graft kyphotic angle, and postoperative complications.

### Data extraction and quality assessment

Two researchers (G.H.L and C.Q.Q) independently screened the literature by reading the titles and abstracts. They reviewed the full texts of relevant studies to determine the final studies that met the inclusion criteria. Discrepancies between the two reviewers were resolved through discussion and consultation with a third reviewer (W.F.F). This study extracted the following information: (1) Basic information of the studies, including title, first author, publication date, and country; (2) Baseline characteristics of the study subjects, including design, sample size, age, and gender; (3) Intervention measures; (4) Clinical indicators.

The Newcastle-Ottawa Scale (NOS) [21] was used as a tool to assess the quality of control clinical studies. This scale consists of 8 items in three broad categories, totaling up to 9 points (denoted by stars): selection of the study groups (4 points), comparability of the groups (2 points), and ascertainment of the exposure (3 points). Generally, a study scoring 6 points or more is considered to be of high quality; otherwise, it is regarded as low quality (Table 1). We employed the Grading of Recommendations Assessment, Development, and Evaluation (GRADE) approach [22] to evaluate the evidence grades of the data for each indicator, as shown in Table 2.

**Table 1:** Score distribution of quality assessment based on Newcastle-Ottawa scale

Items	Yin et al. [10]	Wu et al. [23]	Zhong et al. [24]	Suya et al. [25]	Gao et al. [26]	Koptan et al. [27]	Du et al. [28]	Zhang et al. [29]
**Selection**								
Is the case definition adequate	☆	☆	☆	☆	☆	☆	☆	☆
Representativeness of the cases	☆	☆	☆	☆	☆	☆	☆	☆
Selection of controls	☆	☆	☆	☆	-	☆	-	☆
Defnition of controls	☆	☆	☆	☆	☆	☆	☆	☆
**Comparability**								
Study controls for the most important factor	☆	☆	☆	☆	☆	☆	☆	☆
Study controls for any additional factor	☆	☆	-	-	☆	-	-	-
**Exposure**								
Ascertainment of exposure	☆	☆	☆	☆	☆	☆	☆	☆
Same method of ascertainment for cases and controls	☆	☆	☆	☆	☆	☆	☆	☆
Non-response rate	-	-	-	-	-	-	-	-
**Total scores**	8	8	7	7	6	7	6	7

**Table 2:** Grading of Recommendations, Assessment, Development, and Evaluations

No of studies	Certainty assessment						Effect		Certainty
	Study design	Risk of bias	Inconsistency	Indirectness	Imprecision	Other considerations	No of individuals	Rate (95% CI)	
surgical duration									
8	observational studies	not serious	not serious	not serious	not serious	none	523	-28.06 to 13.67	⨁⨁⨁⨁ High
intraoperative blood loss									
8	observational studies	not serious	not serious	not serious	not serious	none	523	-40.62 to 73.06	⨁⨁⨁⨁ High
graft fusion time									
4	observational studies	not serious	not serious^a^	not serious	Serious^b^	none	231	-0.88 to 2.81	⨁⨁⨁◯ Moderate
ASIA(E)									
5	observational studies	not serious	not serious	not serious	not serious	none	288	0.97 to 1.09	⨁⨁⨁⨁ High
VAS score									
7	observational studies	not serious	Serious^b^	not serious	not serious	none	487	-5.23 to -3.24	⨁⨁⨁◯ Moderate
lumbar pain score									
3	observational studies	not serious	not serious	not serious	not serious	none	155	-45.54 to -34.96	⨁⨁⨁⨁ High
post-graft kyphotic angle									
4	observational studies	not serious	Serious^c^	not serious	not serious	none	303	-36.50 to -6.89	⨁⨁⨁◯ Moderate
postoperative complications									
6	observational studies	not serious	Serious^b^	not serious	not serious	none	429		⨁⨁⨁◯ Moderate

Explanations

a. Apart from three studies, most of the point estimates were closely clustered and the confidence intervals overlapped most of the time. As such, we did not

rate down for inconsistency

b.The confidence interval is large with respect to the pooled estimate. As such, this was a borderline decision to rate down for imprecision

c. Point estimates were rather sparsely distributed, and confidence intervals only overlapped occasionally

### Statistical analysis

Data were analyzed using StataSE15. Binary variables were described using relative risk (RR) and its 95% confidence interval (CI), while continuous variables were expressed as mean difference (MD) with 95% CI. A *P* ≤ 0.05 was considered statistically significant. For meta-analysis, a random-effects model was used when significant heterogeneity was observed. In the absence of heterogeneity, a fixed-effects model was applied. Heterogeneity was assessed using the *I*^*2*^ statistic, ranging from 0 to 100%. A fixed-effects model was used when *I*^*2*^ < 50%, and a random-effects model was used when *I*^*2*^ > 50%. Sensitivity analyses were performed to evaluate the influence of each individual study on the overall effect sizes. One single study was considered to have an influence on the overall effect size if the point estimate of excluding this study fell outside of the 95% CI of the pooled estimates. The publication bias was examined using Begg’s test, with significant publication bias defined as *P* < 0.05.

## Results

### Characteristics and quality assessment of included studies

**Fig. 1:**
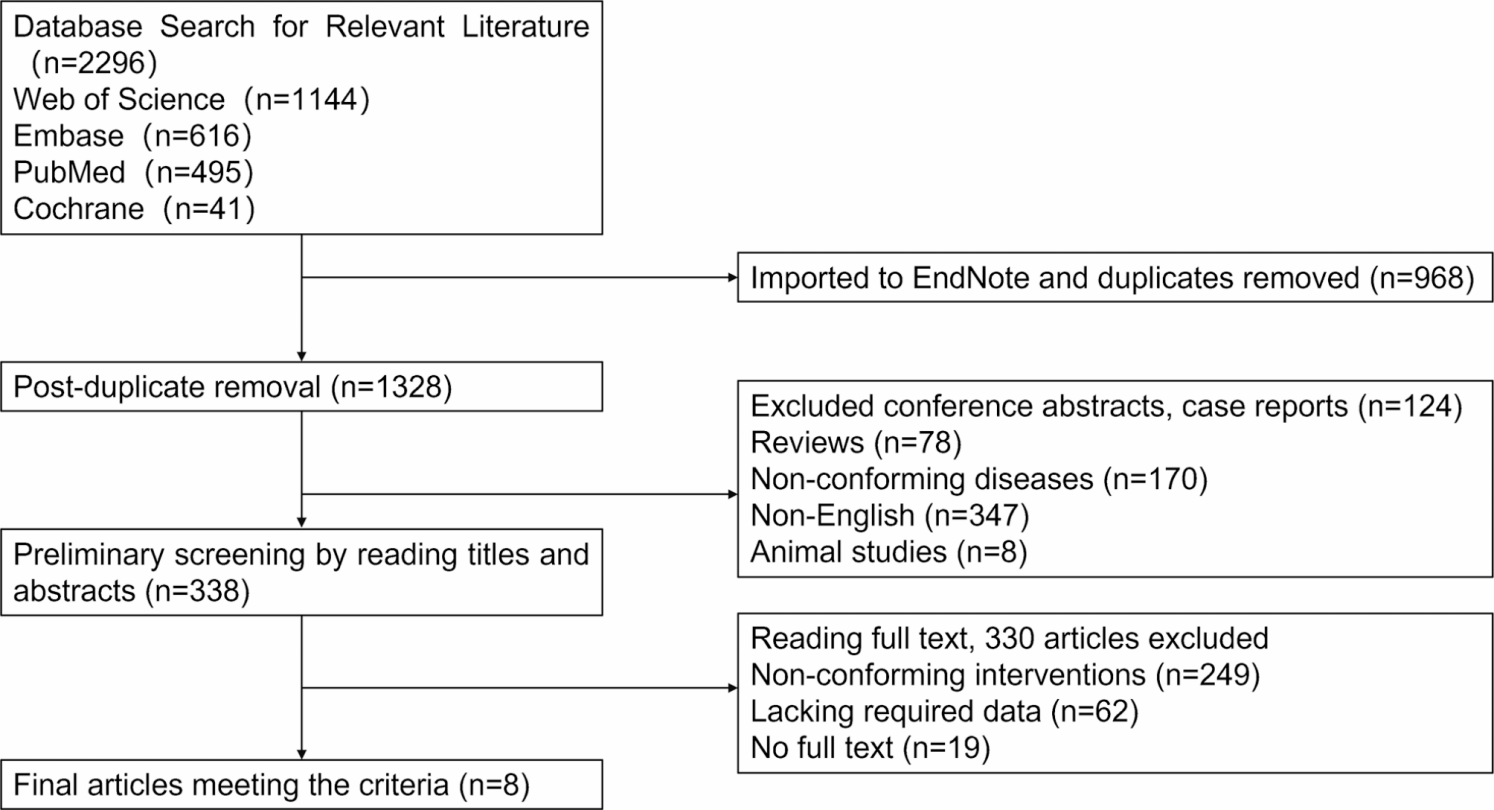
Literature Selection Process

From an initial pool of 2,296 articles, 144 were selected after the first screening. After a thorough review of titles and abstracts, 39 articles remained. After reviewing the full texts of these studies, 8 English articles [10, 23–29] were included in our study (Fig. 1; Table 3), involving 523 cases, with 267 cases of titanium mesh fixation and 256 cases of bone grafting. The general information of patients in all articles was consistent and comparable (Table 4). Seven articles were retrospective studies [10, 23–26, 28, 29], and one was a prospective, non-randomized multi-center study [27]. The quality of these articles was relatively high.

**Table 3:** Basic characteristics of the 8 articles included in this study

Included Literature	Year	Cases		Mean Age		Follow-up Time (months)		NOS Score	Outcome Indicators
		Titanium Mesh	Bone Graft	Titanium Mesh	Bone Graft	Titanium Mesh	Bone Graft		
Wence Wu [23]	2019	86	60	53.1 ± 18.8	48.0 ± 16.5	59.2 ± 17.4	57.5 ± 13.8	8	①②③⑤⑧
Weiyang Zhong [24]	2020	30	30	45.78 ± 19.10	46.23 ± 17.20	48.70 ± 27.30	50.20 ± 25.10	8	①②③④⑤⑥⑦⑧
Danny Suya [25]	2019	19	18	49.53 ± 17.05	56.56 ± 13.00	19.63 ± 4.63	20.33 ± 4.73	7	①②③④⑤⑦
Xin H. Yin [10]	2017	17	19	49.9 ± 15.4	55.5 ± 12.6	46.4 ± 8.0	47.8 ± 8.7	8	①②③④⑥⑧
Yongjian Gao [26]	2017	34	25	39.99 ± 13.65	42.4 ± 12.5	36.09 ± 10.36	34.8 ± 7.0	7	①②⑤⑥⑦⑧
Wael Koptan [27]	2011	16	14	43 ± 13.00	45.57 ± 13.76	65.15 ± 14.70		7	①②⑤⑧
Xing Du [28]	2020	32	66	38.9 ± 13.1	41.45 ± 15.73	28 ± 9.5	27.98 ± 8.58	8	①②③④⑤⑧
Hong-Qi Zhang [29]	2019	33	24	49.03 ± 9.39	48.38 ± 10.75	71 ± 5	74 ± 7	7	①②④⑤⑦

Note: ① Surgical time; ② Intraoperative blood loss; ③ Bone graft fusion time; ④ Spinal cord injury grade E evaluation; ⑤ VAS score; ⑥ Lumbar pain score; ⑦ Kyphotic angle post graft; ⑧ Postoperative complications

**Table 4:** Comprehensive Analysis of Titanium Mesh Grafting versus Bone Grafting for the Treatment of Spinal Tuberculosis

Variable	Cases (Titanium Mesh Grafting)	Titanium Mesh Grafting	Cases (Bone Grafting)	Bone Grafting	*P*	*I²*	WMD/RR (95% CI)	References
Age (years)	267	47.3401 ± 16.6921	256	46.618 ± 15.4691	0.548	7.9	-0.77 (-3.26, 1.73)	10, 21, 22, 23, 24, 25, 26, 27
Operative time (min)	267	205.688 ± 68.7804	256	219.7454 ± 69.6865	0.499	89.7	-7.20 (-28.06, 13.67)	10, 21, 22, 23, 24, 25, 26, 27,
Blood loss (ml)	267	578.949 ± 438.6188	256	579.3316 ± 564.277	0.576	65.4	16.22 (-40.62, 73.06)	10, 21, 22, 23, 24, 25, 26, 27
Bone graft fusion time (months)	98	7.7406 ± 4.8264	133	6.3605 ± 2.4961	0.304	80.4	0.97 (-0.88, 2.81)	10, 22, 23, 26
ASIA (Grade E)	131	122	157	144	0.346	0	1.03 (0.97, 1.09)	10, 22, 23, 26, 27
VAS (Pre-op)	250	5.0137 ± 2.4644	237	5.2054 ± 2.3908	Titanium mesh grafting 0	Titanium mesh grafting 97.3	-4.23 (-5.23, -3.24)	21, 22, 23, 24, 25, 26, 27
VAS (Last follow-up)		1.4017 ± 1.8914		1.6093 ± 1.8107	Bone grafting 0	Bone grafting 96.4	-4.14 (-5.11, -3.17)	
ODI (Pre-op)	81	54.8736 ± 15.4809	74	51.2784 ± 12.6148	Titanium mesh grafting 0	Titanium mesh 94.1	-40.25 (-45.54, -34.96)	10, 22, 24
ODI (Last follow-up)		11.9511 ± 8.6174		12.627 ± 10.1113	Bone grafting 0	Bone grafting 85.3	-36.64 (-40.17, -33.12)	
Kyphotic angle (Pre-op)	116	30.5019 ± 19.1637	97	24.0738 ± 10.4981	Titanium mesh grafting 0	Titanium mesh grafting 97.9	-21.69 (-36.50, -6.89)	22, 23, 24, 27
Kyphotic angle (Last follow-up)		11.6174 ± 13.3071		13.5738 ± 12.7622	Bone grafting 0	Bone grafting 98.3	-10.84 (-18.36, -3.32)	
Postoperative complications	215	64	214	73	0.643	62	0.87 (0.49,1.55)	10, 21, 22, 24, 25, 26

### Meta-analysis results

#### Comparison of surgery duration between titanium mesh grafting and bone grafting

**Fig. 2:**
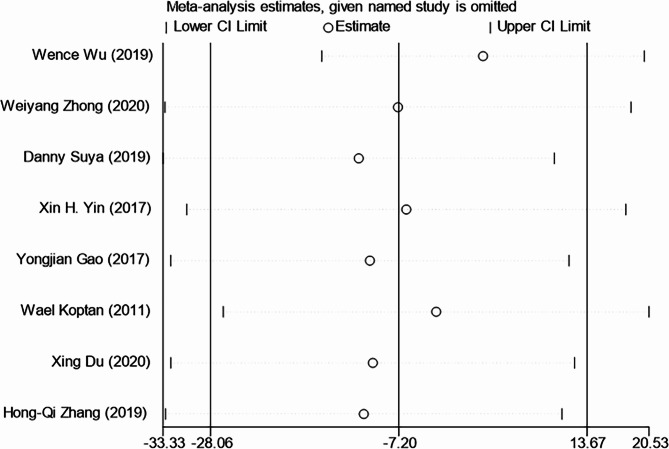
Sensitivity Analysis of Surgery Duration between Titanium Mesh Grafting and Bone Grafting

**Fig. 3:**
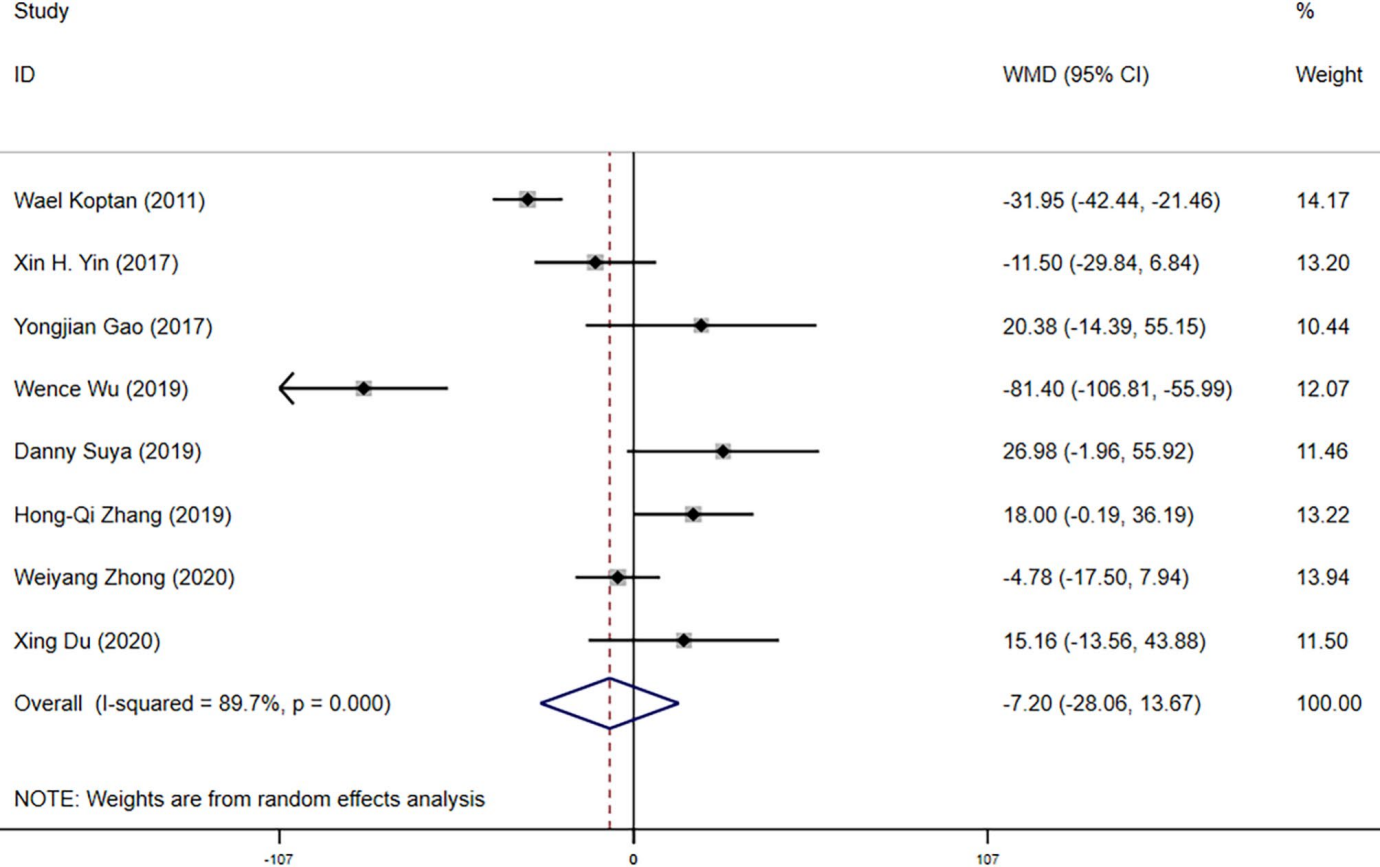
Forest Plot Comparing Surgery Duration between Titanium Mesh Grafting and Bone Grafting

The 8 included articles compared the surgery durations of the two transplantation methods [10, 23–29]. Heterogeneity tests revealed *P* = 0.000 and *I*^*2*^ = 87.9%. A sensitivity analysis of the included literature using a one-by-one exclusion method indicated stable research results, as shown in Fig. 2 The publication bias test demonstrated no significant bias (Begg’s Test *P* = 0.536). A random-effects model was utilized for analysis. The results revealed that surgery durations for spinal tuberculosis treatment were similar between titanium mesh grafting and bone grafting, with no significant difference (Weighted Mean Difference (WMD= -7.20, 95%CI: -28.06, 13.67, *P* = 0.499) as presented in Fig. 3 This indicates no marked discrepancy in surgical duration between the two.

#### Comparison of intraoperative blood loss between titanium mesh grafting and bone grafting

The 8 included articles [10, 23–29] compared the intraoperative blood loss between the two transplantation methods. The heterogeneity test of the literature showed *P* = 0.005 and *I*^*2*^ = 65.4%. Sensitivity analysis, presented in Fig S1, was performed using a one-by-one article exclusion method. The combined effect size remained statistically significant, and the structure of the forest plot did not change noticeably, suggesting that the meta-analysis results are stable and reliable. Additionally, variations in surgical duration can also affect intraoperative blood loss. There was no significant publication bias (Begg’s Test *P* = 0.536). A random-effects model was used for analysis. The results demonstrated that there was no significant difference in intraoperative blood loss when treating spinal tuberculosis with titanium mesh grafting compared to bone grafting. The difference was not statistically significant (WMD = 16.22, 95%CI: -40.62, 73.06, *P* = 0.576), as shown in Fig S2.

#### Comparison of bone fusion time between titanium mesh grafting and bone grafting

Four articles [10, 23–25, 28] compared bone fusion time. The literature heterogeneity test showed *P* = 0.002 and *I*^*2*^ = 80.4%. Sensitivity analysis indicated stable results. A random-effects model was employed for analysis. The findings suggested that there was no significant difference in bone fusion time when treating spinal tuberculosis using titanium mesh grafting compared to bone grafting (WMD = 0.97, 95%CI: -0.88, 2.81, *P* = 0.304), as presented in Fig S3.

#### Comparison of Grade E spinal cord injury assessment between titanium mesh grafting and bone grafting

Five articles [10, 24, 25, 28, 29] compared the Grade E spinal cord injury assessment. No heterogeneity was found between the results of each study, with *P* = 0.990 and *I*^*2*^ = 0%. Analysis with the fixed-effects model revealed that the Grade E assessment of spinal cord injury for spinal tuberculosis treatment was comparable between titanium mesh grafting and bone grafting, with no significant difference (RR = 1.03, 95% CI: 0.97, 1.09, *P* = 0.346), as shown in Fig S4 Both methods exhibited a similar recovery ratio, implying that either type of internal fixation can provide favorable motor sensation recovery in patients.

#### Comparison of VAS score between titanium mesh grafting and bone grafting

Seven articles [23–29] compared preoperative VAS scores and last follow-up VAS scores. A heterogeneity test showed significant heterogeneity between the studies (Titanium Mesh *I*^*2*^ = 97.3%, Bone Grafting *I*^*2*^ = 96.4%). Both were analyzed using the random-effects model. The results indicated that the VAS scores before and after surgery for both titanium mesh grafting and bone grafting were significantly different, as shown in Fig S5 and Fig S6 Both grafting methods can effectively relieve persistent local pain caused by the invasion of *Tuberculous bacilli* into the vertebrae. Further analysis showed that even though the 95% CI of both methods overlapped and the difference was not statistically significant.

#### Comparison of ODI lumbar pain score between titanium mesh grafting and bone grafting

Three articles [10, 24, 26] compared ODI scores. When comparing the lumbar pain scores before surgery and at the last follow-up for spinal tuberculosis treated with titanium mesh grafting, there was heterogeneity among the studies (*I*^*2*^ = 94.1%, *I*^*2*^ = 85.3%). Analysis using the random-effects model showed significant differences in the last follow-up lumbar pain scores between titanium mesh grafting and bone grafting with *P* = 0.000, as shown in Fig S7 and Fig S8 The 95% CI of the two groups overlapped, indicating no significant difference.

#### Comparison of postoperative kyphosis angle between titanium mesh grafting and bone grafting

Four articles [24–26, 29] compared the postoperative kyphosis angle. Both titanium mesh grafting and bone grafting were compared before surgery and at the last follow-up. Significant heterogeneity was found between the studies *I*^*2*^ = 97.9%, *I*^*2*^ = 98.3%). A random-effects model was used for analysis. The result showed significant differences in the postoperative kyphosis angle between preoperative and last follow-up for titanium mesh grafting (WMD= -21.59, 95% CI: -32.19, -10.92, *P* = 0.000), as shown in Fig S9, and for bone grafting (WMD= -11.68, 95%CI: -16.80, -6.55, P = 0.000), as shown in Fig S10 Both grafting methods significantly corrected the postoperative kyphosis angle. Upon further analysis, the 95% CI for both titanium mesh grafting and bone grafting overlapped, indicating no statistically significant difference between the two.

#### Comparison of overall complication rates

A total of 6 articles reported on complications [10, 23, 24, 26–28], encompassing a diverse range of complications such as implant subsidence, cerebrospinal fluid (CSF) leakage, and wound infection. The total sample size was 428 cases, with 215 cases in the titanium mesh grafting group and 213 cases in the bone grafting group. There were 64 cases of complications (29.76%) in the titanium mesh grafting group and 73 cases (34.27%) in the bone grafting group. Heterogeneity tests suggested significant heterogeneity among the studies (*P* = 0.022, *I*^*2*^ = 62.0%). Sensitivity analysis, after individually excluding articles and subsequent combined analysis, revealed no significant shift in heterogeneity. Thus, a random-effects model was employed. The results demonstrated no significant difference in the incidence of complications between the two groups [RR = 0.87, 95% CI: 0.49, 1.55, *P* = 0.643], as seen in Fig S11.

### Subgroup analysis by study type

(1) Implant Subsidence: Two articles reported on postoperative implant subsidence, with a total of 206 patients included. Heterogeneity tests indicated *P* = 0.208, *I*^*2*^ = 37%, implying low inter-study heterogeneity. A fixed-effects model was used for analysis. The results indicated a significant difference in postoperative implant subsidence rates between titanium mesh grafting and bone grafting (RR = 9.30, 95% CI: 1.05, 82.22, *P* = 0.045), as seen in Fig S2. This indicates that the rate of postoperative implant subsidence was slightly lower in bone grafting compared to the titanium mesh grafting group.

(2) CSF Leakage: Three articles reported on postoperative CSF leakage, incorporating 303 patients. Heterogeneity tests showed *P* = 0.669, *I*^*2*^ = 0%, suggesting no heterogeneity among studies. A fixed-effects model was used for analysis. The results demonstrated no significant difference in postoperative CSF leakage rates between titanium mesh grafting and bone grafting (RR = 2.18, 95%CI: 0.58, 8.19, *P* = 0.248), as illustrated in Fig S13.

## Discussion

Our study compared the safety and effectiveness of titanium mesh and bone grafting in the treatment of spinal tuberculosis. The results indicate that both grafting methods are effective in treating spinal tuberculosis, with no significant differences in surgical process and postoperative indicators. This is inconsistent with previous studies comparing these two grafting methods.

In spinal tuberculosis, the primary goals of surgical intervention are threefold: (1) thorough removal of tuberculous lesions to expedite the pathological recovery of the affected area; (2) alleviation of neural and spinal cord compression to save nerve function; (3) correction of local deformities and reconstruction of spinal stability. The selection of materials for reconstructing the spinal column structure is a matter of contention. Currently, widely used bone grafting materials in clinical settings mainly include autografts (rib, iliac bone) and allografts [11]. Autografts are considered the “gold standard” for spinal grafting due to their readily accessible nature, superior biocompatibility, and exceptional osteoinductive, osteoconductive, and osteogenic capabilities [17]. However, studies by Hu and Li have demonstrated that harvesting autografts requires management of the donor site. This approach to autograft bone transplantation consequently prolongs the duration of surgery, increases blood loss at the donor site, and may lead to potential complications at the donor site [30, 31]. However, this meta-analysis shows no statistical difference in surgical duration and intraoperative blood loss between titanium mesh grafting and bone grafting, which could be attributed to factors such as the length of the surgical segment, the amount of bone needed for filling, and the surgeon’s technique.

Bone fusion is an indicator of spinal stability. WANG et al. [32] conducted a one-stage posterior lesion removal, titanium mesh cage grafting, posterior internal fixation, and fusion treatment on 15 older patients with multilevel non-contiguous spinal tuberculosis from September 2009 to October 2013. After an average follow-up of 40 months, all patients were cured of spinal tuberculosis and achieved graft fusion, with no significant angle loss at the last follow-up. ZHANG et al. [29] performed debridement, intervertebral grafting, posterior internal fixation, and fusion surgery on 57 patients with thoracolumbar spinal tuberculosis and kyphotic deformity from January 2011 to March 2013. They used allografts to reconstruct the anterior column and titanium mesh cages, and after a 5-year follow-up, they concluded that compared to allograft bone grafting for thoracolumbar spinal tuberculosis, titanium mesh grafting achieved better spinal stability and great bone fusion. However, this study found no statistical differences in bone fusion time and Cobb angle correction, indicating that both grafting methods can safely achieve final fusion. This is because all included studies combined grafting with internal fixation, suggesting that both grafting methods can achieve good postoperative stability.

In terms of complications, this study shows that there are no statistical differences in overall complications between the two grafting methods. However, bone grafting showed a lower incidence rate of postoperative implant subsidence compared to titanium mesh grafting. HUR et al. [33] suggested that severe subsidence after titanium mesh grafting can lead to poor spinal nerve function recovery, vertebral instability, and reconstruction failure, making it crucial to implement effective interventions to prevent postoperative titanium mesh subsidence. Research conducted by JI et al. [34] explored various factors that could influence postoperative titanium mesh subsidence. Their findings revealed that both optimizing the placement of titanium mesh and starting anti-osteoporosis treatment 6 months preoperatively can help reduce postoperative titanium mesh subsidence.

Although current research shows no statistical differences in surgical-related and clinical outcomes between the two grafting methods, more doctors are inclined to choose titanium fusion devices as the preferred option for vertebral reconstruction in spinal tuberculosis. This preference can be attributed primarily to the limitations of drug therapy, which often struggles to achieve effective medication concentration in the lesion area without causing adverse effects on other organs [35, 36]. Studies have shown that titanium mesh grafting has better biocompatibility and can rapidly stabilize the affected segments without causing pathology at the grafting site, making it widely used in the clinical treatment of spinal tuberculosis [9, 16, 37]. Meanwhile, simple bone grafting has its limitations, such as limited autologous bone quantity and potential complications from donor site bleeding and potential complications in the graft area [38]; allografts are associated with complications such as delayed healing and infection [39]. Therefore, titanium mesh grafting not only avoids the pain and infection of the autograft harvest site but also allows for extensive biological integration at the bone interface with titanium mesh. Additionally, the adjustable length and strong support capability of titanium mesh have also been recognized by many clinicians.

However, this does not imply that bone grafting is inferior. Our data analysis does not support this viewpoint either. Numerous studies have shown that patients with spinal tuberculosis are predominantly found in economically underdeveloped developing countries and impoverished regions [6, 40, 41]. Due to limitations in medical resources and economic constraints, patients in these areas may not be able to choose titanium mesh as the grafting material. Therefore, for regions with insufficient medical resources or underdeveloped economic conditions, bone grafting remains the only viable option. Our study provides a basis for clinicians to choose the most suitable grafting material for their patients based on local insurance policies and the patients’ financial capabilities.

Our meta-analysis has several strengths. It encompasses a comprehensive systematic review, incorporating the most recently published cohort studies on titanium mesh grafting versus bone grafting. The analysis includes studies characterized by high methodological quality, with scores ranging from 6 to 8. Additionally, the quality of evidence was assessed using the Evaluations (GRADE) approach, allowing for the confirmation of the consistency and robustness of the primary results.

Moreover, over the past 20 years, China has been listed by the WHO as one of the countries with a high burden of tuberculosis globally [42]. With a large number of patients with spinal tuberculosis, a significant volume of surgical samples, and a variety of graft fusion surgical methods, studies from China represent a substantial portion of the research in this field. Although the majority of the studies we collected were from China, the results still retain scientific validity and representativeness.

Currently, this study has several limitations. Firstly, all 8 studies we included were non-randomized controlled experiments. Non-randomized controlled trials are susceptible to bias or confounding factors [43]. Despite the good quality of each included study, the nature of non-randomized controlled trials forces us to acknowledge that the risk of bias remains high. Secondly, some studies had small sample sizes, making it difficult to determine differences in outcomes for some complications, such as non-union and recurrence rates. Therefore, it was not possible to compare these outcomes in our data analysis. Thirdly, while considering treatment methods, various systemic treatments were utilized, but the focus of this study was primarily on surgical treatment. Therefore, drug types were not within the scope of our discussion. Additionally, among the 8 studies, only one involved the cervical spine [27]. Excluding its results, the overall direction of the results remained unchanged, as shown in Table S1. Our results are applicable to thoracic, lumbar, and cervical regions. However, given the limited research data on the cervical spine, further research is needed to confirm these conclusions. Lastly, many studies did not provide detailed records of the location and number of affected segments in spinal tuberculosis, preventing subgroup analysis based on different locations and segments. Furthermore, since the included literature did not differentiate between granular bone grafting or block bone grafting, a systematic analysis of the advantages of granular bone grafting could not be conducted, which is a major cause of heterogeneity.

## Conclusion

In summary, both simple bone grafting and titanium mesh grafting are effective and safe for the surgery, with no significant differences in the statistical results. This does not support previous studies that suggested the superiority of titanium mesh grafting. Since the meta-analysis of non-randomized controlled trials carries a risk of bias, and due to the small sample sizes for some complications preventing statistical analysis, conducting randomized controlled trials is expected to further test the reliability of these results.

### Electronic supplementary material

Below is the link to the electronic supplementary material.

Supplementary Material 1

Supplementary Material 2

## Data Availability

The datasets used and/or analyzed during the current study available from the corresponding author on reasonable request.

## Abbreviations

ASIA: American Spinal Injury Association
VAS: Visual analogue scale
ODI: Oswestry Disability Index
CSF: Cerebrospinal Fluid
