# Supplementary material for: Effectiveness and safety analysis of titanium mesh grafting versus bone grafting in the treatment of spinal Tuberculosis: a systematic review and meta-analysis
Source: BMC Surg. 2023 Dec 12;23:377. doi: 10.1186/s12893-023-02283-1 (PMC10717474; doi:10.1186/s12893-023-02283-1)
Supplement: Supplementary file 1 — Supplementary Material 1 [file 12893_2023_2283_MOESM1_ESM.docx]

**Supplementary Figures**

**
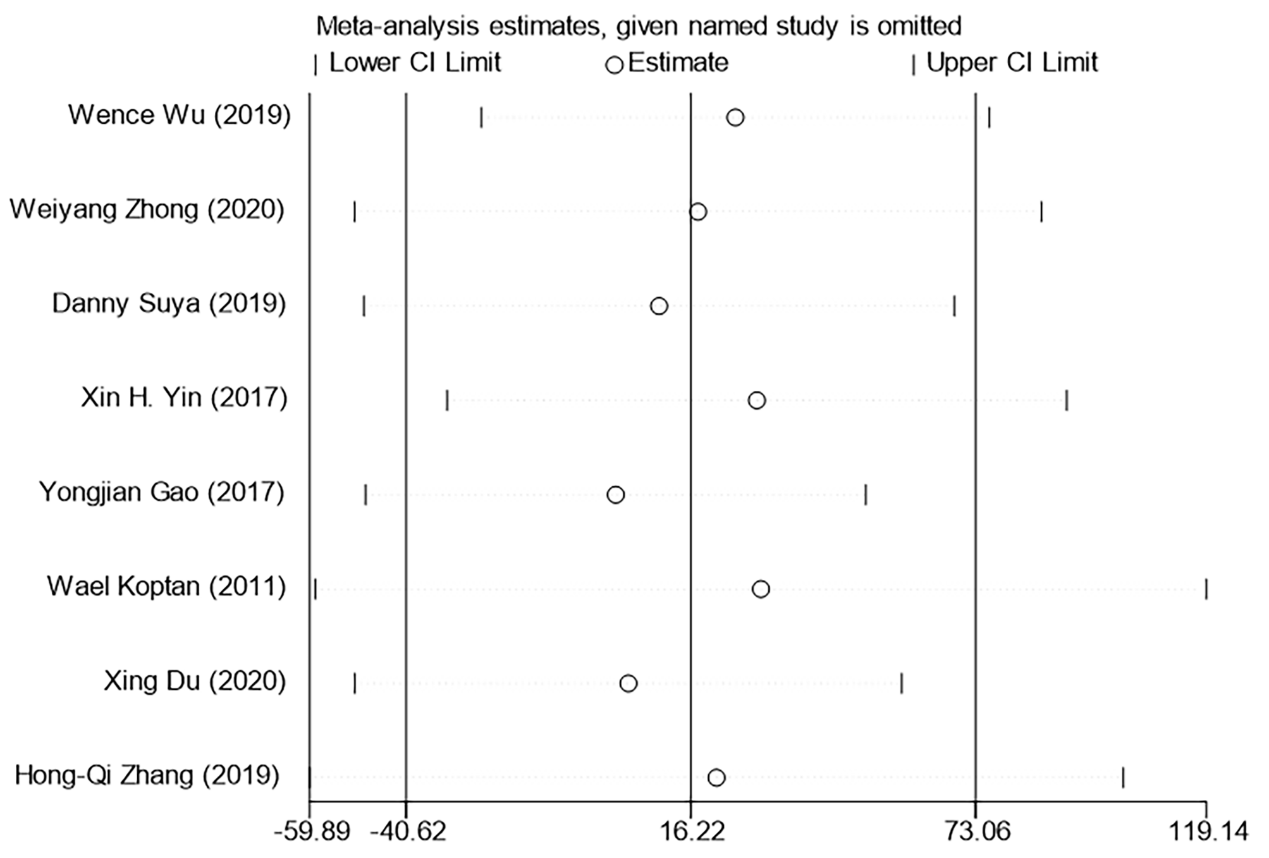
**

**Fig S1** Sensitivity Analysis of Blood Loss Comparison between Titanium Mesh Grafting and Bone Grafting

**
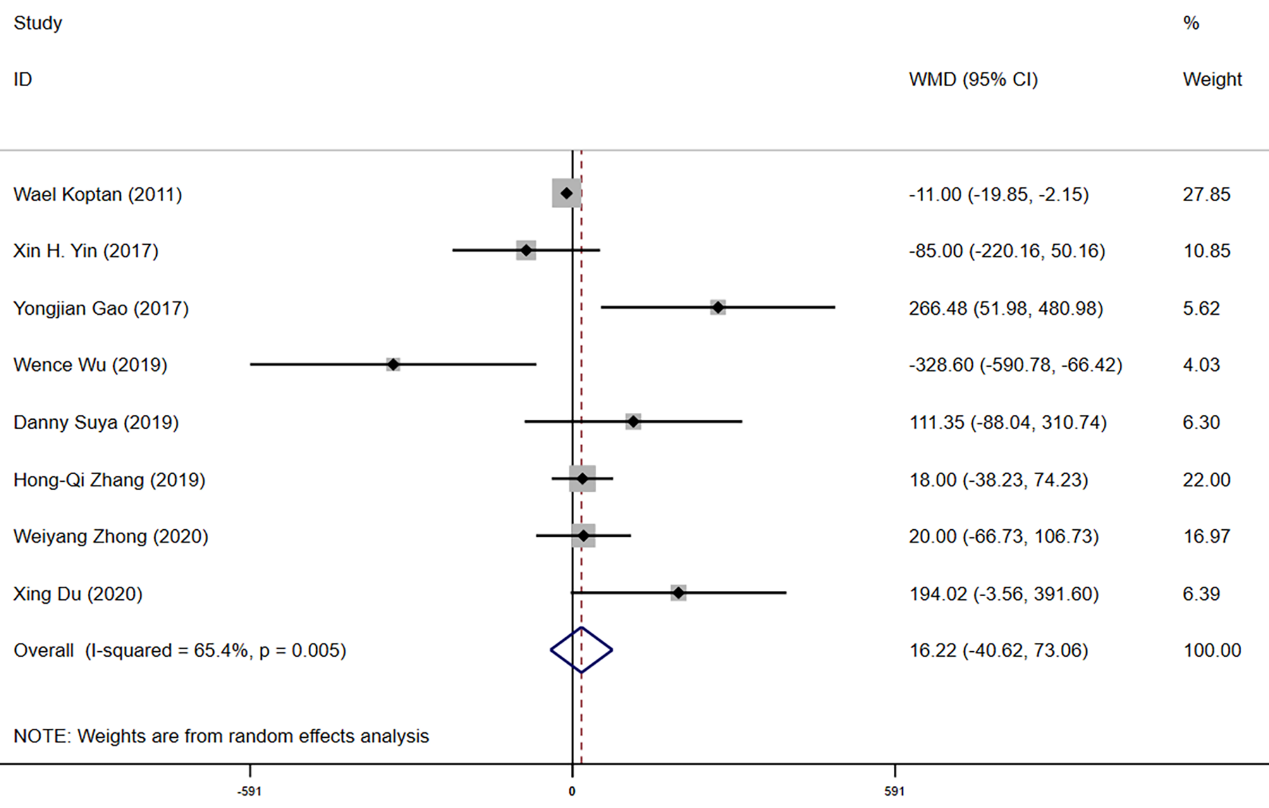
**

**Fig S2** Forest Plot of Blood Loss Comparison between Titanium Mesh Grafting and Bone Grafting

**
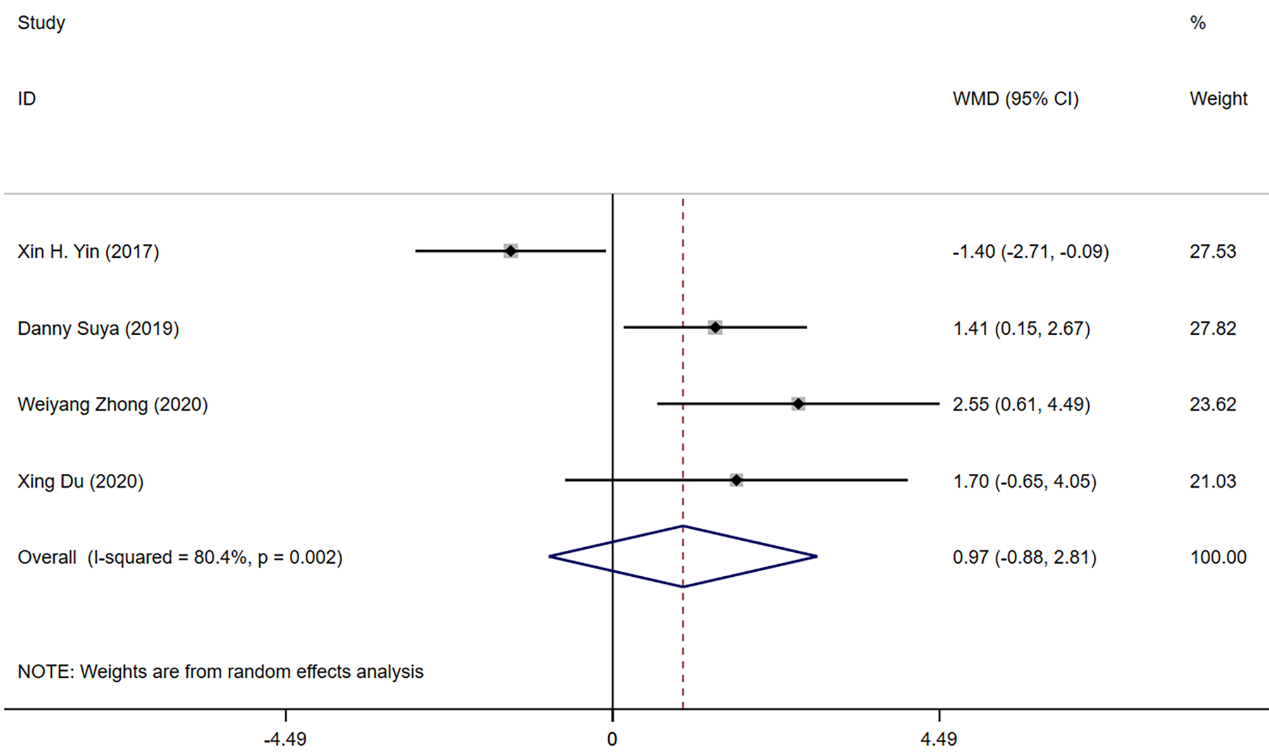
**

**Fig S3** Forest plot comparing bone fusion time between titanium mesh grafting and bone grafting

**
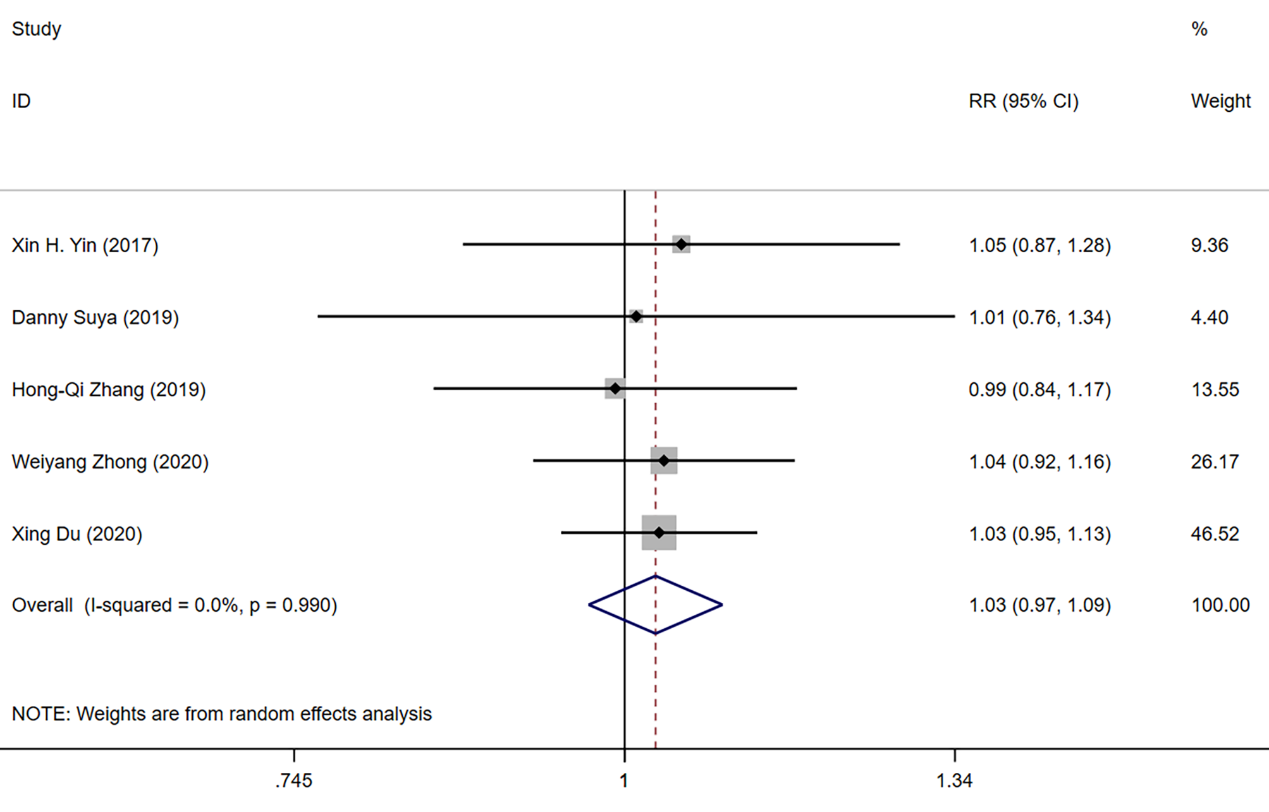
**

**Fig S4** Forest Plot Comparing the Grade E Spinal Cord Injury Assessment Between Titanium Mesh Grafting and Bone Grafting

**
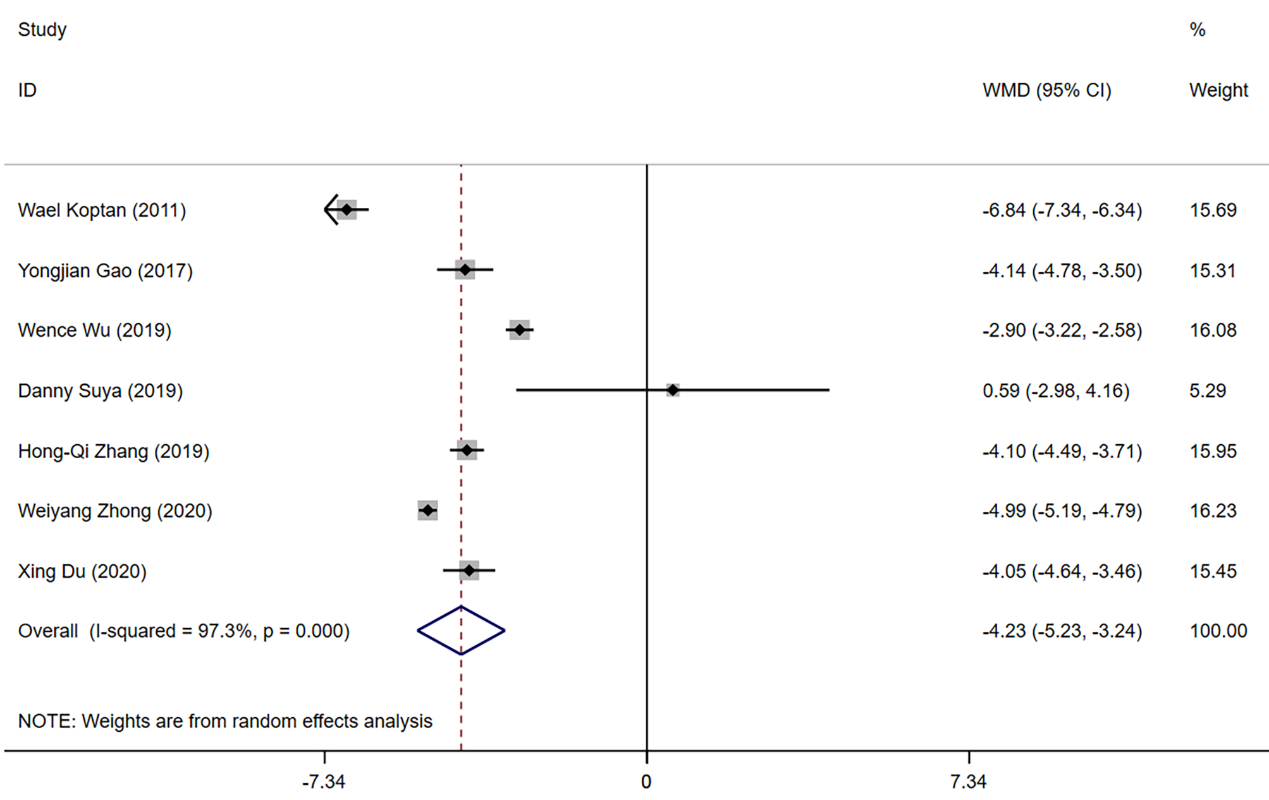
**

**Fig S5** Forest Plot Comparing the VAS Assessment Before Surgery and at Last Follow-up for Titanium Mesh Grafting

**
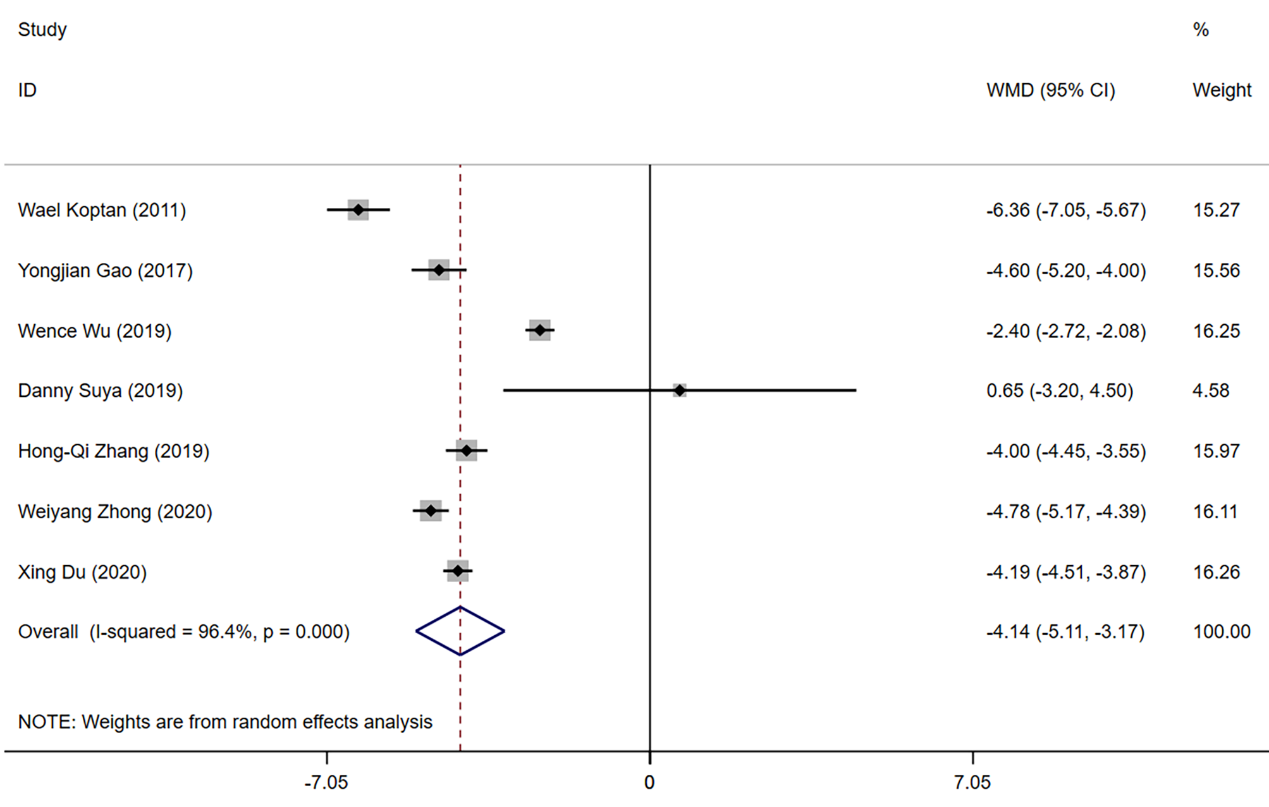
**

**Fig S6** Forest Plot Comparing the VAS Assessment Before Surgery and at Last Follow-up for Bone Grafting

**
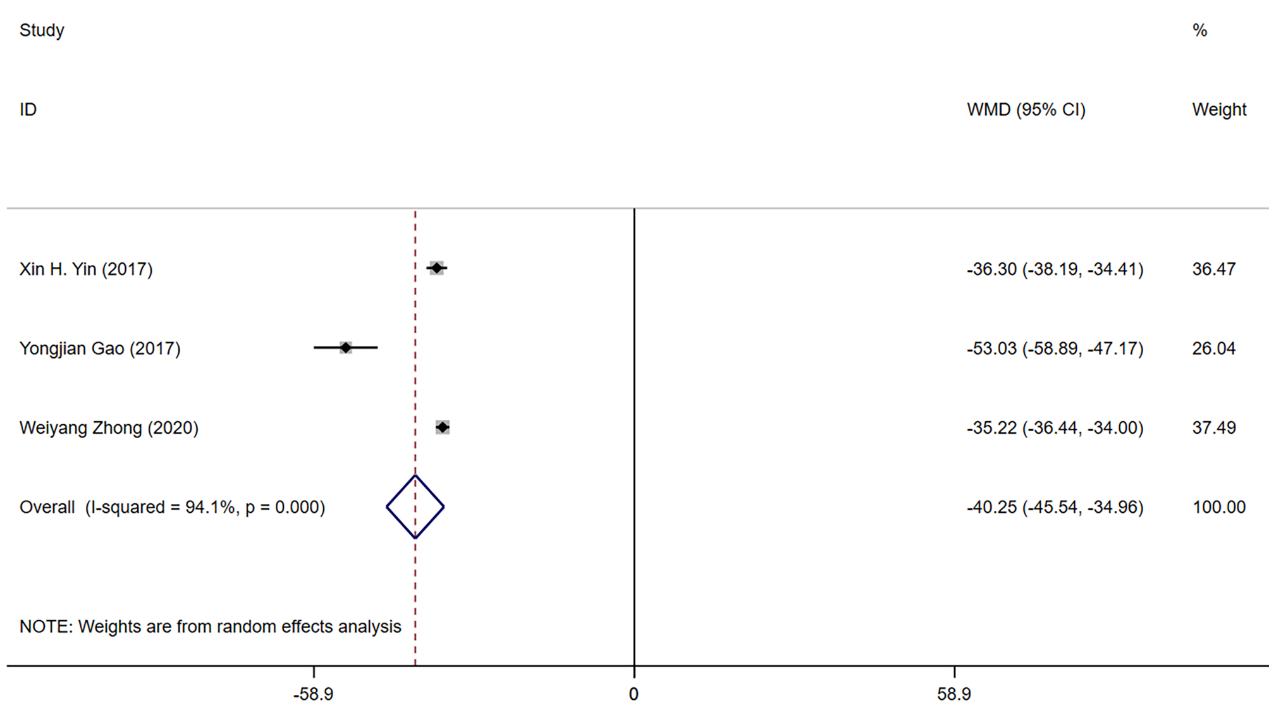
**

**Fig S7** Forest Plot Comparing Pre-surgery and Last Follow-up ODI Scores after Titanium Mesh Grafting

**
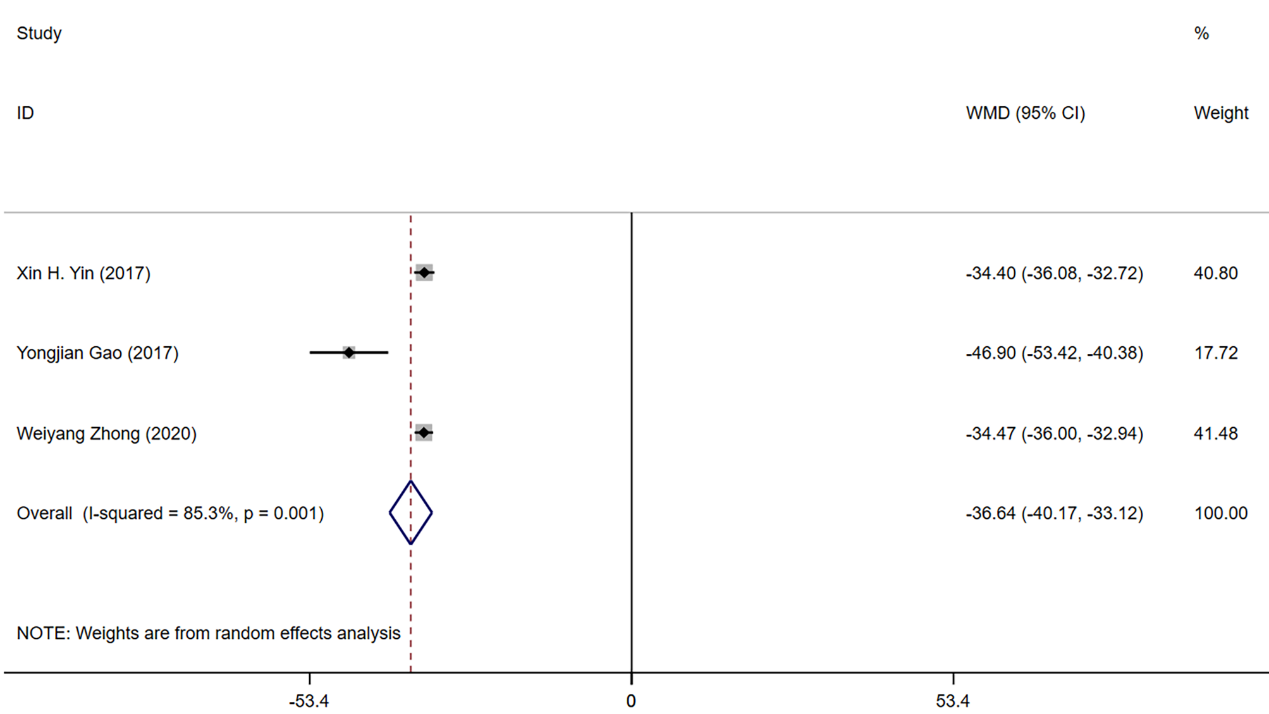
**

**Fig S8** Forest Plot Comparing Pre-surgery and Last Follow-up ODI Scores after Bone Grafting

**
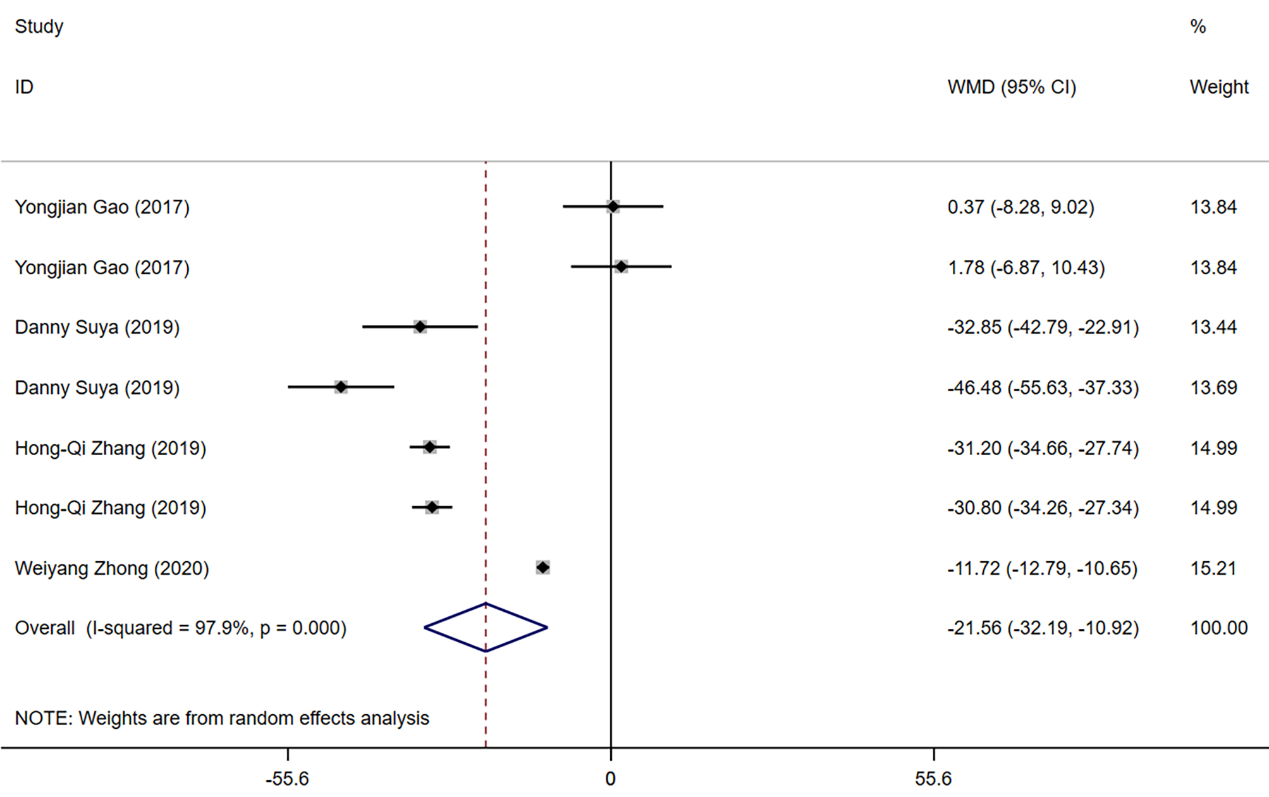
**

**Fig S9** Forest Plot Comparing the Preoperative and Last Follow-up Kyphotic Angle after Titanium Mesh Grafting

**
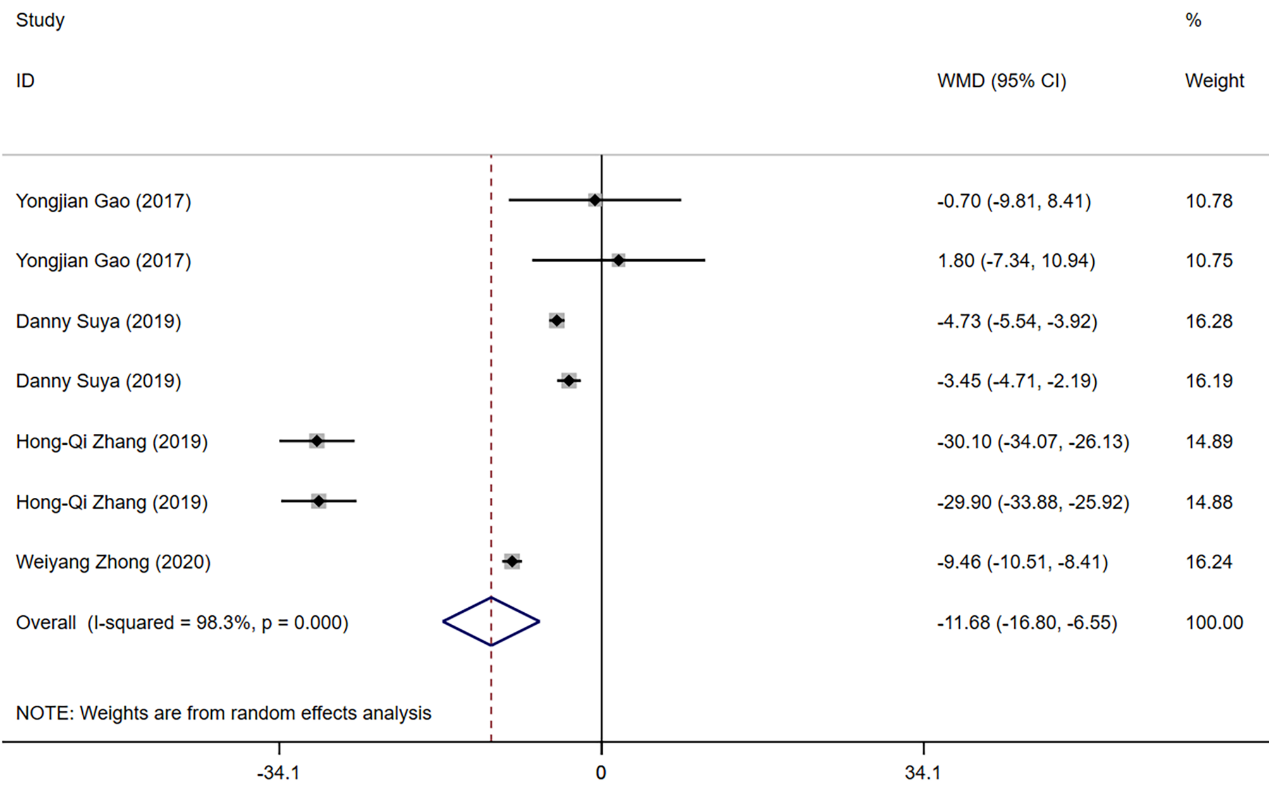
**

**Fig S10** Forest Plot Comparing the Preoperative and Last Follow-up Kyphotic Angle after Bone Grafting

**
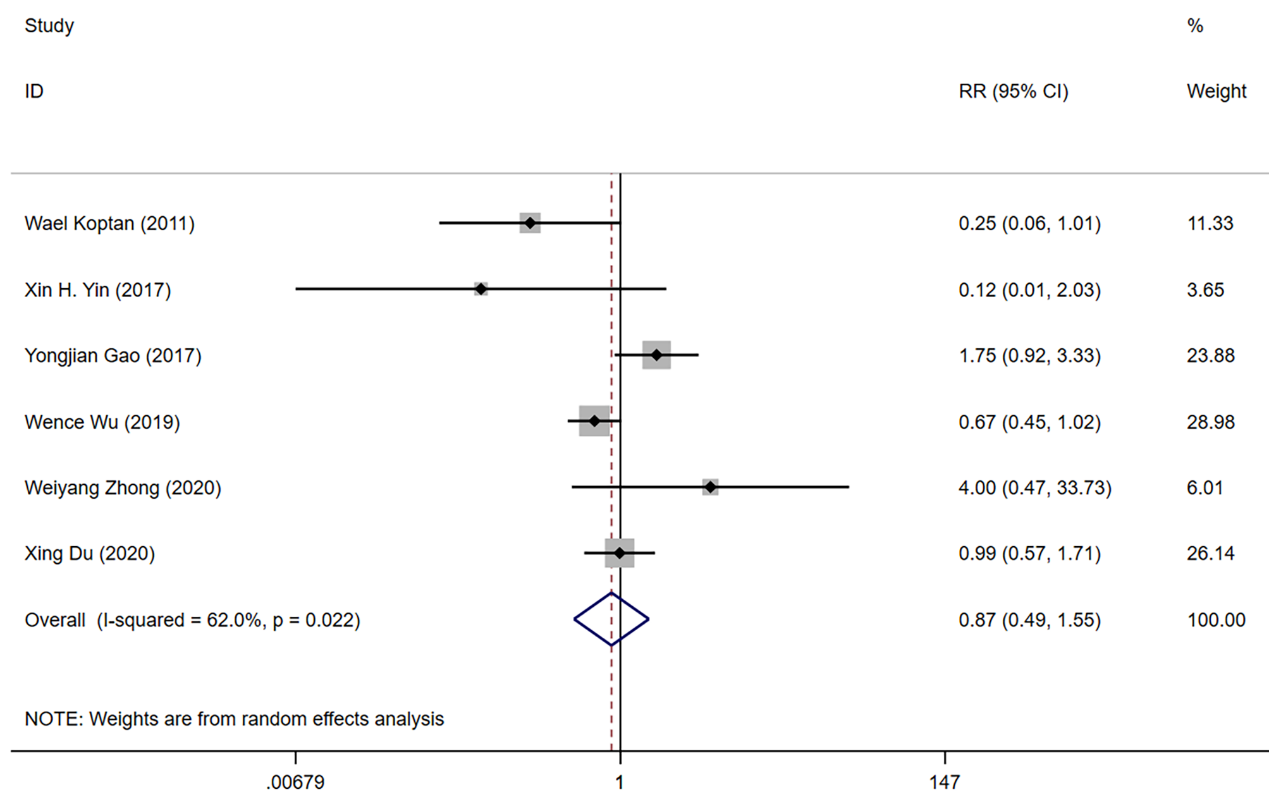
**

**Fig S11** Forest Plot Comparing the Overall Complication Rates Between the Titanium Mesh Grafting Group and the Bone Grafting Group

**
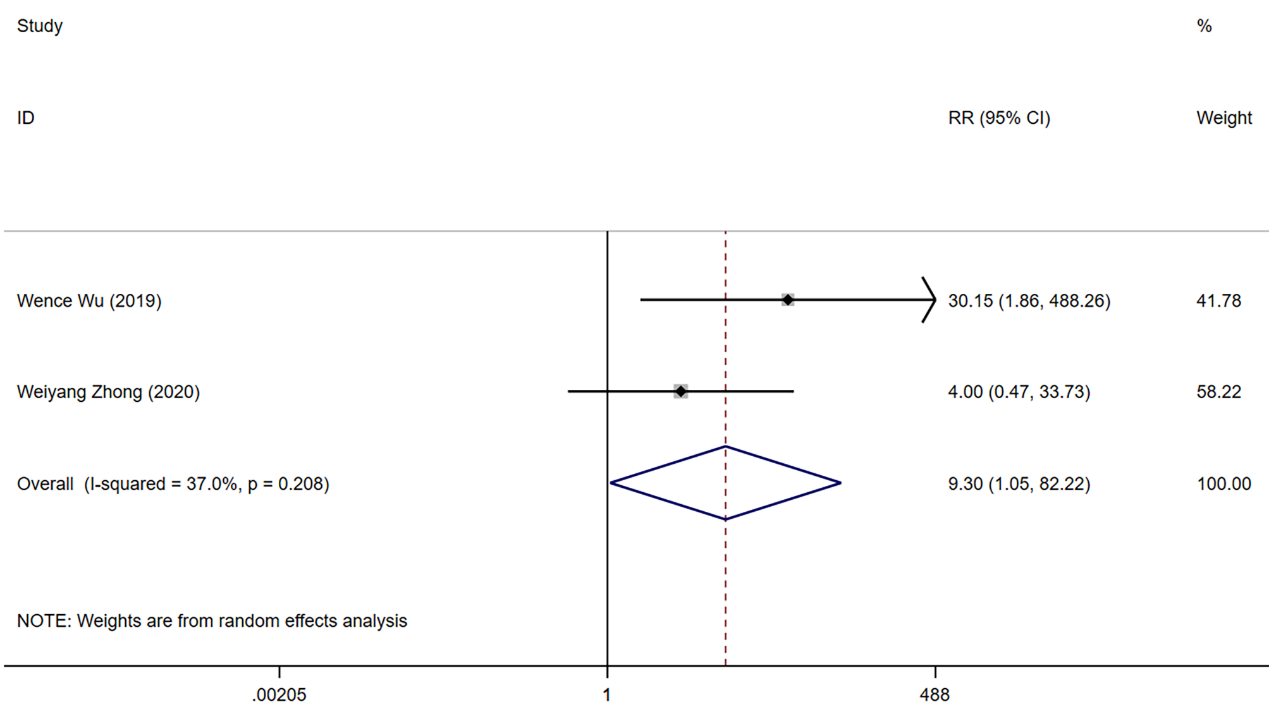
**

**Fig S12** Forest Plot Comparing the Incidence of Implant Subsidence Between the Titanium Mesh Grafting Group and the Bone Grafting Group

**
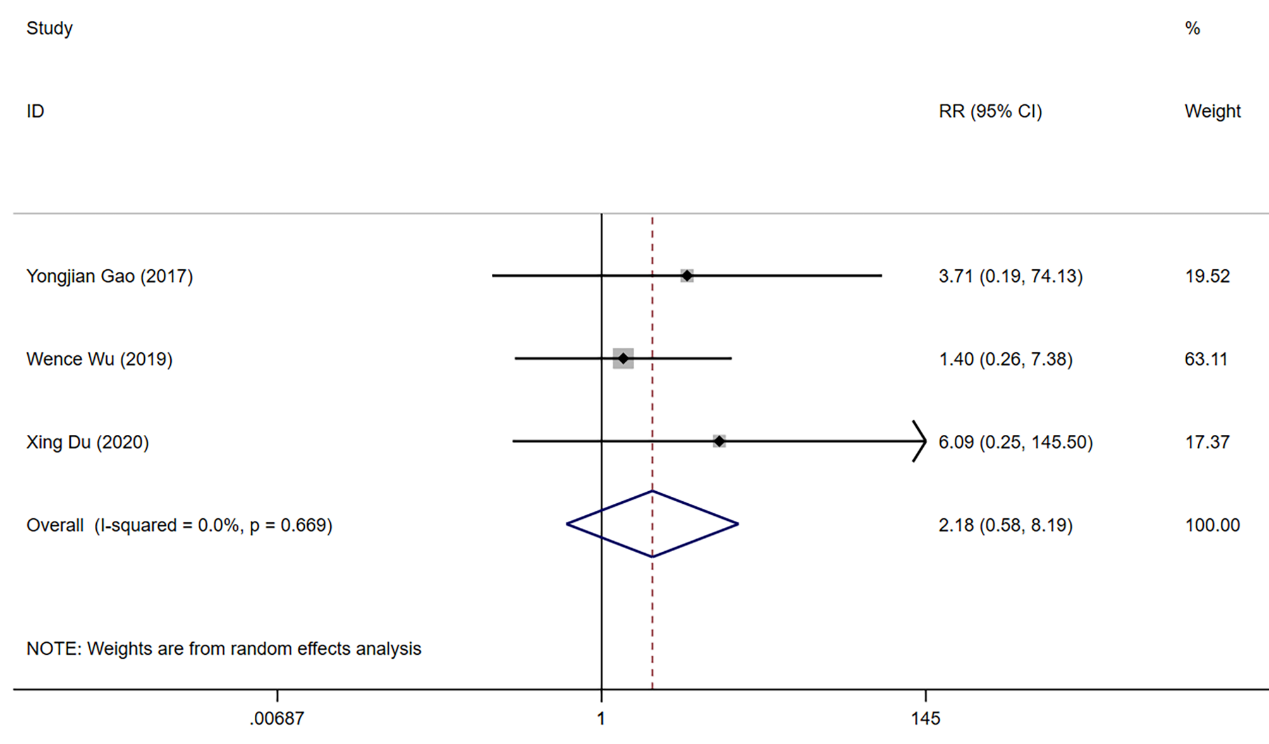
**

**Fig S13** Forest Plot Comparing the Incidence of CSF Leakage Between the Titanium Mesh Grafting Group and the Bone Grafting Group

**Table S1** Data comparison of 95% CI after excluding ref.27

| Variable | WMD/RR (95% CI) | 95% CI after excluding ref.27 | References |
| --- | --- | --- | --- |
| Age (years) | -0.77 (-3.26, 1.73) | -3.76, 2.11 | 10, 23, 24, 25, 26, 27，28，29 |
| Operative time (min) | -7.20 (-28.06, 13.67) | -26.65, 20.53 | 10, 23, 24, 25, 26, 27，28，29 |
| Blood loss (ml) | 16.22 (-40.62, 73.06) | -58.76, 119.14 | 10, 23, 24, 25, 26, 27，28，29 |
| VAS (Pre-op) | -4.23 (-5.23, -3.24) | -4.72, -2.91 | 23, 24, 25, 26, 27, 28, 29 |
| VAS (Last follow-up) | -4.14 (-5.11, -3.17) | -4.70, -2.85 |  |
